# Effects of litter and additional enrichment elements on the occurrence of feather pecking in pullets and laying hens – A focused review

**DOI:** 10.1002/vms3.184

**Published:** 2019-07-03

**Authors:** Ruben Schreiter, Klaus Damme, Eberhard von Borell, Isabelle Vogt, Michael Klunker, Markus Freick

**Affiliations:** ^1^ ZAFT e.V. Centre for Applied Research and Technology Dresden Germany; ^2^ Bayerische Staatsgüter Lehr‐, Versuchs‐ und Fachzentrum für Geflügel‐ und Kleintierhaltung Kitzingen Germany; ^3^ MLU Halle‐Wittenberg – University Halle Germany; ^4^ HTW Dresden – University of Applied Sciences Dresden Germany

**Keywords:** barn housing, beak trimming, egg production, plumage damage

## Abstract

Severe feather pecking (SFP) is a serious problem in the egg production industry with regard to animal welfare and performance. The multifactorial causes of SFP are discussed in the areas of genetics, feeding, husbandry, stable climate and management. Several studies on the influence of manipulable material on the incidence of SFP in different environments and housing systems have been performed. This review presents current knowledge on the effects of litter and additional enrichment elements on the occurrence of SFP in pullets and laying hens. Because SFP is associated with foraging and feed intake behaviour, the provision of manipulable material in the husbandry environment is an approach that is intended to reduce the occurrence of SFP by adequate exercise of these behaviours. As shown in the literature, the positive effect of enrichment and litter substrate on SFP in a low‐complexity cage environment is evident. On the other hand, consistent results have not been reported on the influence of additional enrichment material in housing systems with litter substrate, which represent the most common type of husbandry in Northwestern Europe. Thus, further research is recommended.

## Abstract 2

Severe feather pecking (SFP) is a serious problem in the egg production industry with regard to animal welfare and performance. This review presents current knowledge on the effects of litter and additional enrichment elements on the occurrence of SFP in pullets and laying hens. While the positive effect of enrichment and litter substrate on SFP in a low complexity cage environment is evident, consistent results have not been reported on the influence of additional enrichment material in housing systems with litter substrate.

## Graphical abstract

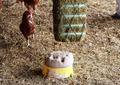

## 1 INTRODUCTION

**Figure 1:**
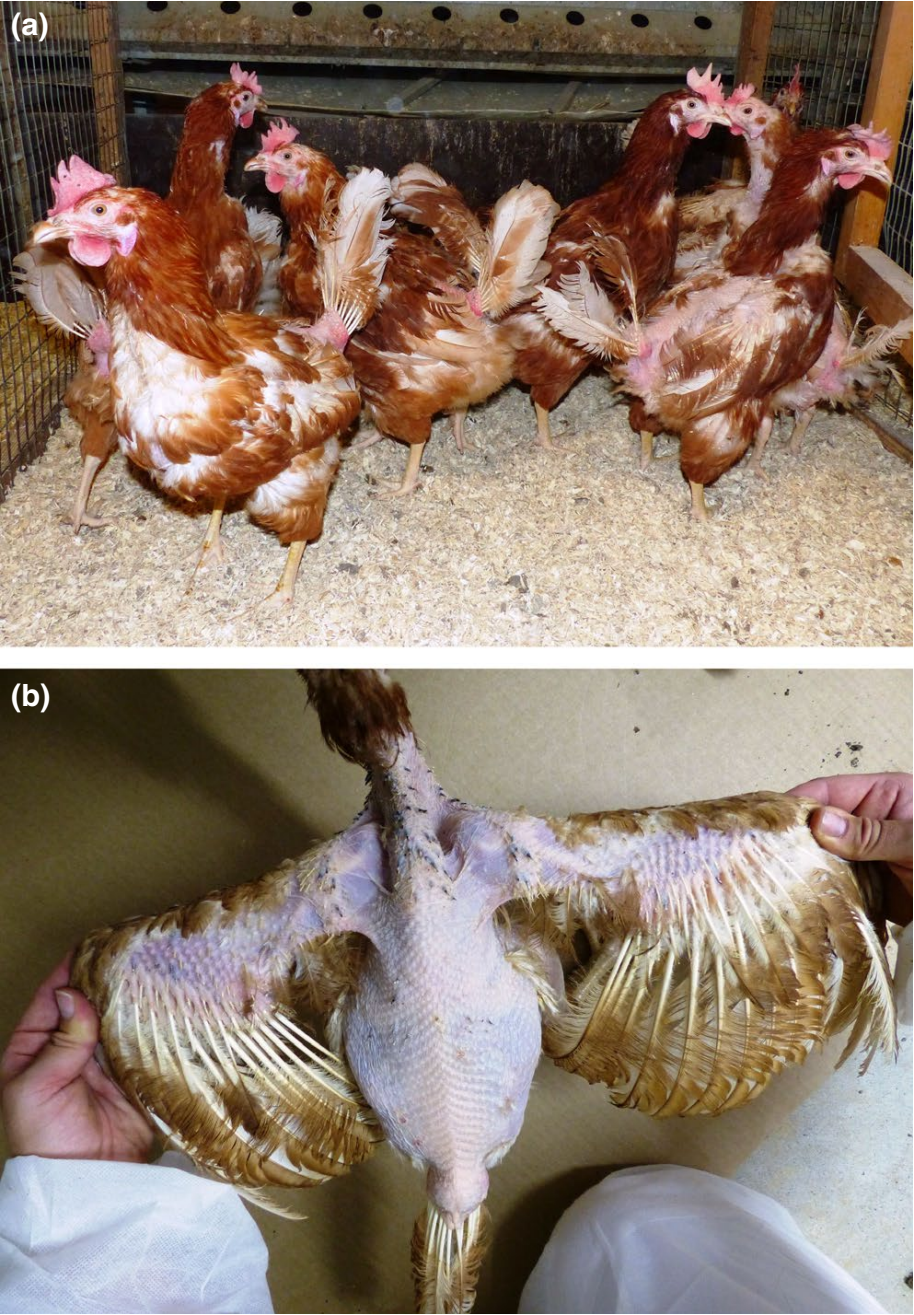
The risk of severe feather pecking and plumage damage (a, b) has increased by keeping laying hens with untrimmed beak tips in various European countries (a) (Sepeur et al., 2015)

Feather pecking (FP) is a serious problem in laying hen husbandry with regard to animal welfare and performance (Appleby & Hughes, 1991; Niebuhr et al., 2006; Rodenburg et al., 2013). Oettel (1873) described this behavioural disorder, which is not a new problem, more than 100 years ago. Perpetrator hens peck feathers or parts of feathers of conspecifics, whereupon the feathers may also be eaten (Rodenburg et al., 2013). Like cannibalism, FP is not an aggressively motivated behavioural disorder, and two forms of FP can be differentiated: gentle FP (GFP) and severe FP (SFP) (Savory, 1995). GFP is considered a normal exploratory behaviour, whereas SFP leads to plumage damage and featherless areas, which can promote cannibalism and associated skin injuries (Rodenburg et al., 2013; Savory, 1995). The occurrence of SFP in a flock can lead to impaired animal welfare, as extensive feather loss significantly restricts the well‐being of the hens (Rodenburg et al., 2013), and the pecked animals suffer from pain (Gentle & Hunter, 1991). However, SFP is not only a problem of animal welfare but is also disadvantageous from the view of production because of further undesirable consequences, such as increased mortality, lower laying performance and increased feed consumption due to the increased energy demand in case of plumage loss (Damme & Pirchner, 1984; El‐Lethey, Aerni, Jungi, & Wechsler, 2000; Niebuhr et al., 2006; Wechsler, Huber‐Eicher, & Nash, 1998) (Figure 1).

The causes of SFP are multifactorial, concerning the areas of genetics, feeding and management (Bessei, Lutz, Kjaer, Grashorn, & Bennewitz, 2018; Kjaer & Bessei, 2013; Van Krimpen, Kwakkel, Van der Peet‐Schwering, Den Hartog, & Verstegen, 2008). Beak trimming commonly has been used to reduce the negative effects of SFP over a long period (Damme, 1999; Spindler, Giersberg, Andersson, & Kemper, 2016). However, because this non‐curative intervention has been examined more critically, and several North‐western European countries have begun to avoid beak trimming, the risk of SFP has increased significantly (Sepeur et al., 2015). In addition to breeding measures to reduce the genetic predisposition to behavioural disorders, the optimization of the husbandry environment and feeding are central areas for minimizing the incidence of SFP (Kjaer & Bessei, 2013; Rodenburg et al., 2013; Spindler et al., 2016). Feather pecking is considered as misdirected foraging and feed intake behaviour (Blokhuis, 1986; Gilani, Knowles, & Nicol, 2013; Wennrich, 1975), where the phenotypic patterns of SFP are similar to those of feed and ground pecking (Dixon, Duncan, & Mason, 2008). In this respect, the provision of litter and other changeable materials is particularly important for the reduction of SFP (Rodenburg et al., 2013).

The aim of this study was to present current knowledge on the effects of litter and additional enrichment elements on the occurrence of SFP in pullets and laying hens. An additional contribution of this review is to identify gaps in knowledge and research needs, especially with regard to barn housing, which is the most common husbandry system in Northwestern Europe.

The corresponding literature search was conducted using the databases PubMed (https://www.ncbi.nlm.nih.gov/pubmed/) and Google Scholar (https://scholar.google.de/) with the following keywords: “laying hens AND feather pecking AND litter OR substrate OR foraging OR floor” or “laying hens AND feather pecking AND enrichment”. Scientific papers were included if the effects of litter and/or enrichment elements on FP in pullets and/or laying hens housed in cage or barn systems were investigated in experimental or field studies and if a control group was implemented in the study. In Germany, stabling of beak‐trimmed pullets has been abandoned since 2017 by a voluntary agreement between the poultry industry and the Federal Government (BMEL 2015). Since then, research activities have increased in national or regional projects. Thus, the search results of these projects and recommendations from German agricultural and veterinary authorities for the husbandry of non‐beak‐trimmed laying hens have been included in this review if the studies considered enrichment elements and devices.

Investigations to analyse the influence of manipulable material on the incidence of SFP have taken place in different environments and animal husbandry systems. In particular, distinctions must be made between experiments carried out on wired floors without litter (cage systems) or whether the animals were kept in an environment with litter and, thus, within conditions of alternative housing regarding the presence of a floor substrate. Most previous investigations on the influence of manipulable material on SFP compared litter‐free systems on perforated floors (cages or enriched cages) to husbandry on different litter substrates but did not examine the effect of additional enrichment material in housing systems with litter.

## 2 EFFECTS OF LITTER

**Figure 2:**
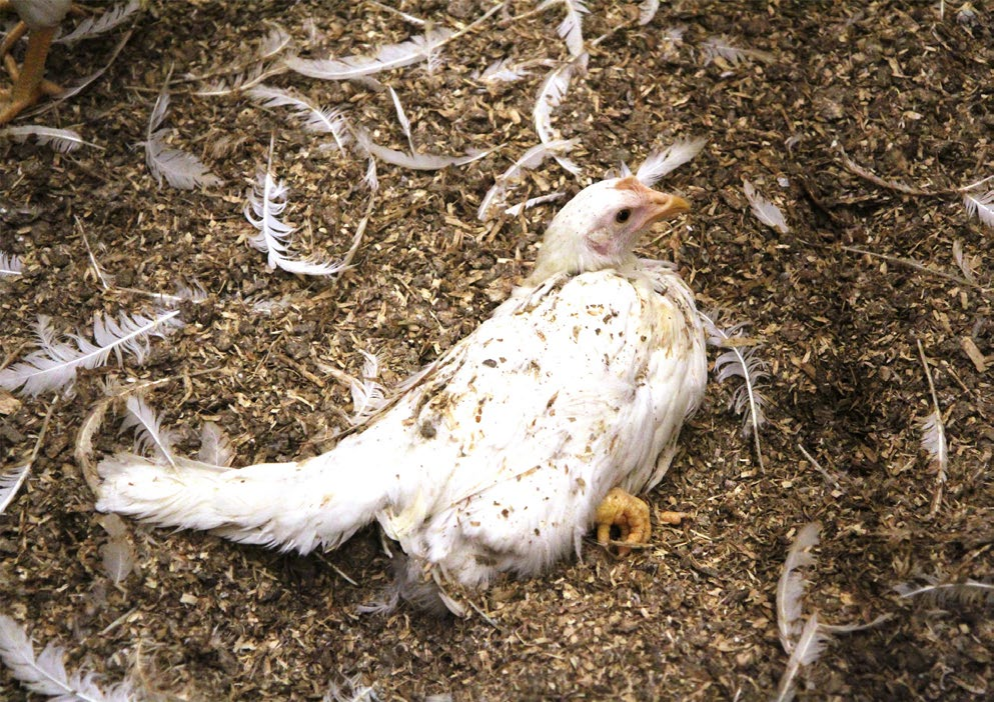
Early access to litter can reduce feather pecking in comparison to housing on wire floors (Johnsen et al., 1998)

In a series of studies, the presence of manipulable material was shown to improve the plumage condition in pullets and laying hens (Figure 2). Blokhuis and Van der Haar (1989) investigated the effects of littered floors (wood shavings) or wired floors during rearing and the subsequent laying period. According to this approach, the groups with litter floors showed less SFP and more ground pecking in the rearing period as well as in the later laying period. To identify possible differences in the effects of dust‐bath vs. foraging materials, Huber‐Eicher and Wechsler (1997) reared white‐egg layer chicks on perforated floors with access to sand as a dust‐bath substrate or access to straw as a feed substrate. High SFP rates and injuries were detected in the sand‐bath group but not in the group with access to straw, which showed less SFP but more forage‐seeking behaviour with straw. Therefore, the authors concluded that a suitable feed substrate promotes foraging behaviour and SFP reduction or even delay. Similar results were obtained by Dixon & Duncan, 2010, who also observed that sand‐bath substrates, when provided as the only manipulable material in the husbandry environment, are not able to reduce SFP. In this comparison, chicks were kept on wired floors or on solid floors covered with peat moss. Gilani et al. (2013) identified a higher risk for SFP in the laying period when SFP already occurred during rearing due to insufficient access to manipulable material. In a study by Green, Lewis, Kimpton, and Nicol (2000), the absence of fluffy litter substrate at the end of the laying period was a risk factor for the occurrence of SFP.

With the knowledge that manipulable materials can reduce behavioural disorders, several groups compared the applicability of different litter substrates. Huber‐Eicher and Wechsler (1998) kept chicks on wooden grates and examined the influence of various manipulable substrates, especially foraging materials, on the pecking behaviour during their fourth and fifth week of life. Special attention was paid to the form of the substrates (long cut straw, chopped straw, polystyrene blocks or polysterol beads). Chicks showed less SFP if they had access to long straw in comparison to chopped straw or polystyrene blocks. When comparing polystyrene blocks to polysterol beads, chicks with the latter material showed increased SFP. An area with wood shavings as the dust‐bath area showed no influence on the SFP.

In a study on the preferences of different substrates for pecking, scratching and dust‐bathing, chicks preferred sand to straw and wood shavings to feathers in the first weeks of life (Sanotra, Vestergaard, Agger, & Lawson, 1995). It was also shown that the substrate known from the first weeks of life is preferred later in life if there is a choice among different materials. At the same time, however, these preferences changed with increasing age due to specific experiences. Savory and Mann (1999) could not identify a higher risk for SFP in litter substrates which form a strong contrast in colour to the feather colouration and thus encourage more pecking to litter particles in the plumage.

Considering the effects of litter on behavioural aspects, from what age onwards the substrates are provided to the animals can be decisive. Against the background of the importance of early access to manipulable material, Huber‐Eicher and Wechsler (1997) showed that chicks with access to sand from the 10th day of life showed higher SFP rates than chicks with access to sand from the first day of life. In a study by Johnsen, Vestergaard, and Norgaard‐Nielsen (1998), of those chicks raised on sand, on sand and straw or on wire floors in the first 4 weeks of life, and kept on sand and straw in the following rearing time, the chicks reared on the wire floor showed the most severe plumage damages, increased SFP and higher mortality due to cannibalism. From this, the authors concluded that access to manipulable material in the first 4 weeks of life has a crucial influence on the later occurrence of SFP. In contrast, Nicol et al. (2001) stated that their study results show it is primarily the current substrate access which is decisive, with the experience from the first weeks of life being less important. In that study, the pullets and laying hens kept on wired floors were provided with wood shavings as litter at different ages for different periods. As expected, hens kept permanently on wired floors showed the most severe cases of SFP. However, the fact that laying hens kept on shavings performed more ground pecking and less SFP than hens kept on wired floors was independent of the hens’ previous experience. Huber‐Eicher and Sebö (2001) compared housing on plastic gratings in an aviary rearing system during the first 2 weeks of life with groups that already had access to wood shavings or straw during this period. In the subsequent weeks, all groups had access to litter. In chicks and pullets with litter access from the first day of life, less FP was observed in weeks 5 and 14, and plumage damage was less severe than in the control variant. Also in the field study by Gunnarsson, Keeling, and Svedberg (1999), an early access to litter (up to fourth week of life) was associated with a lower risk of SFP in the later laying period.

The influence of exposure to material for dust‐bathing and foraging in the early rearing period or during the entire rearing period and later laying period on SFP is subject to different findings. This is particularly interesting as chicks in commercial aviary systems are usually housed in closed aviary segments during the first 3 to 5 weeks of life, and only afterwards access to the litter area is allowed (Pottgüter, Schreiter, & Van der Linde, 2018). The wire floor surface in the aviary segments is commonly covered with so‐called chick paper to encourage the chicks to feed with the feed placed on it, to offer the day‐old chicks a better support with their small extremities and, above all, to establish the faecal contact necessary for successful immunization in coccidiosis vaccination (Lohmann Tierzucht 2017; Pottgüter et al., 2018; Thiele, 2008). This chick paper, with the feed particles on it, represents a manipulable material for the employment of chicks (De Jong, Gunnink, Rommers, & Bracke, 2013; Helmer, 2017).

Additionally, the concrete question about whether it may be beneficial to cover wire floors partially with chick paper in commercial rearing facilities was considered. The investigations by De Jong et al. (2013) were subjected to the question of the effects of litterless rearing, which is common in aviary stables during the first weeks of life, on possible behavioural deviations. For this purpose, the chicks (first to third weeks of life) of the experimental groups were kept on sand or wood shavings. The control groups were kept on grids, paper or chick paper. The supply of litter in the early rearing period stimulated ground‐directed pecking. At the age of 4 weeks, the chicks in the experimental groups showed less GFP than the chicks on grids or paper. Later, no clear effects were observed among the different treatments and groups. The plumage scoring at the 40th week of life showed no differences among the groups.

Behavioural observations of chicks kept on wired floors in closed aviary segments by Helmer (2017) detected a reduction in foraging behaviour and an increase in the SFP rate when chick paper was partially or completely removed and if no additional enrichment material was available. In field studies by Tahamtani et al. (2016) and Brantsaeter et al. (2017), rearing chicks on chick paper in the aviary block during the first 5 weeks of life was able to reduce the anxiety reactions of the hens during the laying period (30th week of life) only in certain parameters compared to chicks raised only on wire floors.

## 3 EFFECTS OF ADDITIONAL ENRICHMENT ELEMENTS

In general, the influence of additional enrichment elements was analysed in two kinds of husbandry systems: cage systems with wired floors and barn housing systems with litter. In the case of cages with wired floors, several studies investigated the influence of additional enrichment elements in chick, pullet and laying hen environment. Chicks and pullets kept on wire floors made very intensive use of additional enrichment elements in the form of textile strings, especially if they were offered very early in life (Jones & Carmichael, 1999) and if the strings were white (Jones, Carmichael, & Rayner, 2000). In a trial by McAdie, Keeling, Blokhuis, and Jones (2005), white‐egg laying hens housed in cages were provided with strings permanently from the first day of life, every 4 weeks for 24 hr, permanently from the 16th week of life or not at all. Hens in cages with the supply of strings showed less plumage damage at the 35th week of life compared to the hens without this additional enrichment. Remarkably, no differences were observed in the plumage scoring among the different intensities of supply.

Dixon, Duncan, and Mason (2010) observed the highest attractiveness and most pronounced reduction of SFP in feed materials as enrichment elements when comparing the different materials used for chicks kept on wire floors. Dust‐baths and novel objects had a medium effect. Without any environmental enrichment, SFP was most pronounced. Aerni, El‐Lethey, and Wechsler (2000) tested white‐egg layers with or without access to long straw when feeding pellet or mash fodder. Only those hens fed with pellets and without access to straw were found to have severe plumage damage.

**Figure 3:**
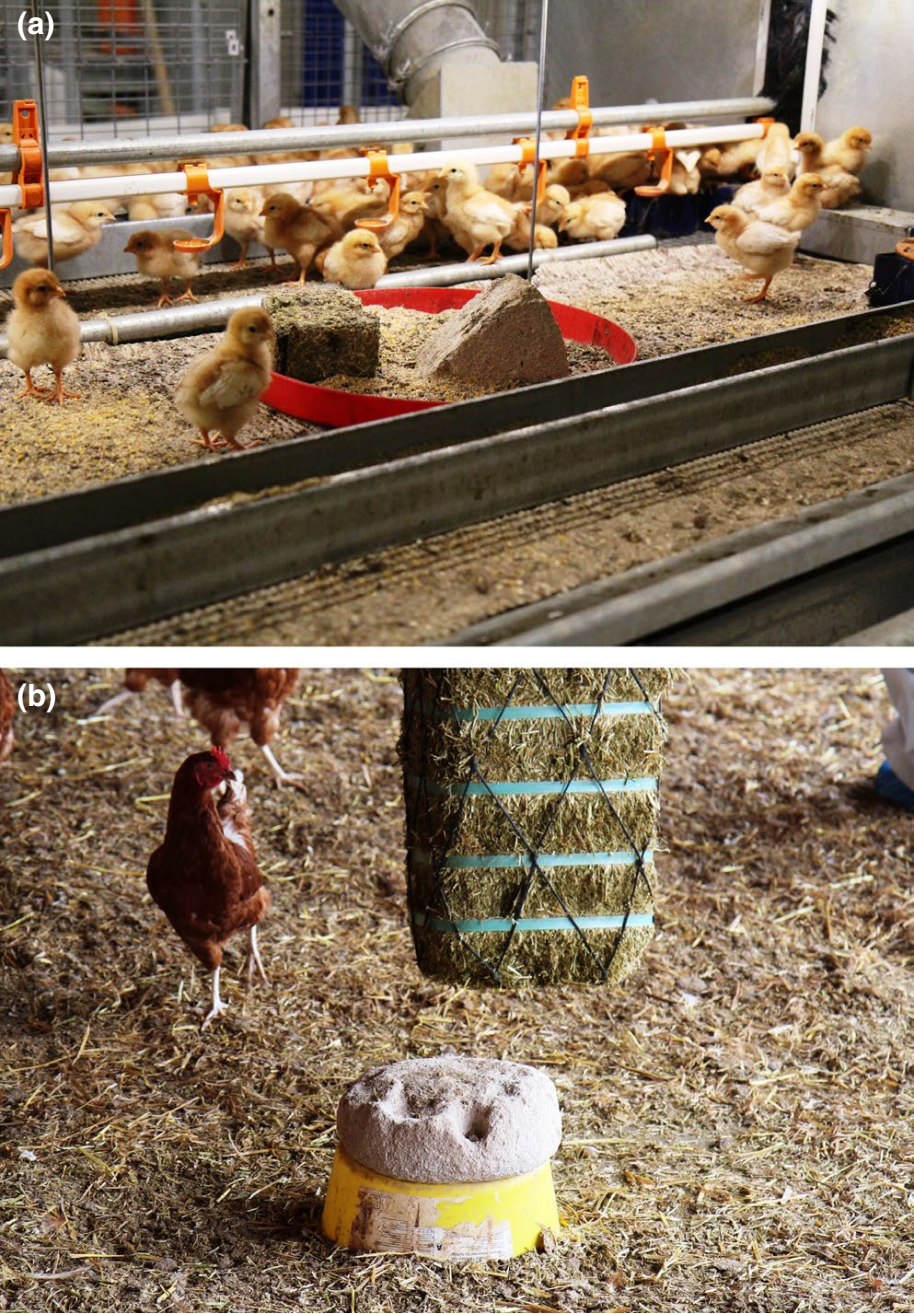
Pecking stones and alfalfa bales are frequently used enrichment materials in chicks (a), pullets and laying hens (b). However, consistent results have not been found regarding the effects of these enrichment materials on the occurrence of feather pecking in housing systems with litter (see Table 1)

In the case of barn housing systems with litter, various authors investigated the effects of the supply of additional enrichment (Table 1, Figure 3). Basically, the applied enrichment materials can be classified into three groups: (a) objects which do not provide the possibility for oral intake or dust‐bathing (e.g. strings), (b) substrates which are suitable for consumption as feed (e.g. straw, alfalfa bales, silages, pecking stones and grain in litter), and (c) installations with substrates for dust‐bathing. Additionally, pecking stones increase the abrasion of the keratin of the upper beak and, therefore, can cause blunting of the tip of the beak (Icken, Cavero, & Schmutz, 2017). Analogous to litter substrates, a graduated aptitude exists for the different enrichment materials. In a study by McAdie et al. (2005), chicks of a white‐egg layer line, which were selected for high SFP, were offered strands of strings as enrichment devices in barn housing. In the period from the first day up to the eighth week, the strongest reduction of SFP was observed when the strings were available permanently or for 4 hr per day. The groups with access to the strings beginning with the 22nd or 52nd day showed an intermediate expression, and the control group without strings showed the highest rate of SFP. To identify differences between these pullets and those pullets reared identically without additional enrichment, the pullets housed in barn housing systems received litter straw or grain (Blokhuis & Van der Haar, 1992). A significant reduction in plumage damage could only be achieved by adding grain to the litter, but not by the addition of straw. The authors concluded that adding grain to the litter during rearing can direct pecking and scratching behaviour to the littered floor and prevent pecking from being redirected to the feathers of other hens during the later laying period. By providing maize silage, pea‐barley silage or carrots as additional enrichment materials to brown‐egg layers housed in a barn housing system, Steenfeldt, Kjaer, and Engberg (2007), could reduce the SFP and plumage damage. Mortality was significantly reduced in the three test variants compared to the control without additional enrichment. The restrictive use of silage and other perishable feeds is a practical consideration on farms, where the simultaneous assurance of animal health has to be considered, especially during rearing because of possible nutritional consequences, along with biosecurity (Steenfeldt et al., 2007). Zepp et al. (2018) housed brown‐layer chicks in aviary housing under production conditions in groups with and without enrichment material (pecking stones and alfalfa bales) at high stocking density and at reduced stocking density. Feather pecking was reduced by offering enrichment material and by reducing the stocking density. Norgaard‐Nielsen, Vestergaard, and Simonsen (1993) determined that white‐layer pullets reared solely on straw showed more SFP in the later laying period than pullets, which in addition to their access to straw, also had access to sand and peat as substrates for dust‐bathing during rearing. Furthermore, in the laying period, the provision of baskets filled with straw as enrichment elements could significantly reduce the increase in plumage damage compared to the control group without this enrichment measure.

**Table 1:** Summary of studies which investigated the effects of additional enrichment materials in littered housing systems on the occurrence of severe feather pecking and plumage damage

Reference	Additional enrichment^a^	Observation period	Genetic strain	Effects on SFP and plumage damage	Assessment
McAdie et al. (2005)	Strings	Chicks/pullets (1st to 57th day of life)	White‐egg layers (Leghorn line, selected on high FP incidence)	Reduction	Positive effects in chicks/pullets
Zepp et al. (2018)	Pecking stones and alfalfa bales	Chicks/pullets (1st to 17th week of life)	Brown‐egg layers (LB)	Reduction	
Blokhuis and Van der Haar (1992)	Straw or grain addition to litter during rearing	Chicks to laying hens (1st to 42th week of life)	Brown‐layers (Warren SSL)	Reduction in laying period by grain addition during rearing	
Norgaard‐Nielsen et al. (1993)	Sand and peat during rearing; straw in baskets during laying period	Chicks to laying hens (1st to 72th week of life)	White‐egg layers (LSL)	Reduction in laying period by both enrichment variants	Positive effects in pullets and laying hens
Steenfeldt et al. (2007)	Maize silage, peas‐barley silage, or carrots	Laying hens (16th to 54th week of life)	Brown‐egg layers (ISA Brown)	Reduction by all enrichment variants	
Hartcher et al. (2015)	Strings, oat in litter, or deeper litter	Chicks to laying hens (1st to 43th week of life)	Brown‐egg layers (ISA brown)	No effects	
Freytag et al. (2016)	Alfalfa bales, pecking stones, grain addition to litter, or pecking stones and grain addition to litter	Chicks to laying hens (1st to 75th week of life)	Brown‐egg layers (LB)	No unidirectional effects of enrichment materials	Lack of consistent effects in pullets and laying hens
Cronin et al. (2018)	Straw	Chicks to laying hens (1st to 40th week of life)	Brown‐egg layers (ISA Brown)	No effect in laying period	

Abbreviations: FP, Feather pecking; LB, Lohmann Brown; LSL, Lohmann Selected Leghorn; ISA, Institute de Sélection Animale.

a The amount of enrichment materials was not reported comparably in all studies. For details, see references.

In addition to the investigations mentioned above, which demonstrate the reducing effect of enrichment materials on SFP, a number of studies were unable to identify the positive effects. Using a two‐factor approach, Hartcher et al. (2015) investigated the influence of enrichment materials and beak trimming in brown‐layers in barn housing. As measures for the additional enrichment in the husbandry environment, strings, an oat supplement in the litter or a deeper litter were used. The plumage condition in the 43rd week of life as an indirect parameter for SFP could not be improved by any of the three enrichment variants compared to the control. In contrast, beak trimming reduced SFP and feather damage.

Remarkably, Lugmair (2009) and Lambton, Knowles, Yorke, and Nicol (2010) found an even higher risk of SFP in flocks with grain added to the litter in their field studies. In another field study, Freytag, Kemper, and Spindler (2016) compared the influence of different enrichment variants (pressed alfalfa bales, pecking stones, grain addition to litter and pecking stones plus grain addition) to controls without additional enrichment during the rearing and laying period for 100,000 brown‐layers housed in aviary systems. Regarding mortality, no effects of the enrichment material were observed in the rearing period. In the laying period, the lowest animal losses were observed in the groups without enrichment material and with grain addition. In all groups with added enrichment material, problems increased with piling up and smothering. The lowest plumage damage was observed in the group with alfalfa bales, and the highest damage was observed with pecking stones or pecking stones plus grain addition. At the same time, integument injuries were less frequent in the group with alfalfa bales than in the other groups. No unidirectional effect was observed in the variants with enrichment material to lower plumage damage and skin injuries compared to the control. Alfalfa bales were used more intensively than the pecking stones by the hens in the scratching room. Cronin et al. (2018) examined the effects of straw as enrichment material in addition to examining re‐stabling stress in brown‐layer pullets and laying hens (1st to 40th week of life) housed in littered compartments with exercise. Therefore, from the sixth week of life onwards, racks were provided with straw, or without straw for the control group. In addition, half of the hens were stimulated with re‐stabling stress in the 16th week of life as a stressor with practical relevance (moving to laying facility). As an effect of the enrichment material, the behavioural observations showed more ground pecking for all phases, whereas a reduction in mutual pecking was only observed in the pullet age but not during the laying period. Access to long straw did not cause any significant change in plumage scores, but plumage losses tended to be lower in groups without straw. Mortality was significantly higher in the straw groups (10.5%) compared to the control group without straw (6.0%). The provision of enrichment material had no effects on body mass, laying performance, the proportion of floor eggs and feed consumption, as well as humidity and pH value of the litter.

## 4 PERMANENT OR SITUATIONAL ACCESS TO ENRICHMENT?

On the basis of scientific findings and, in particular, practical experiences, various recommendations to laying hen farmers regarding the supply of enrichment materials have been published. However, no consensus exists regarding the question about whether enrichment material should be provided permanently as a preventive measure or only from the time when the first signs of SFP occur within the flock. To reduce the risk of SFP, management instructions mostly recommend the preventive supply of enrichment devices, such as pecking stones, hay baskets, alfalfa bales, juice feed or widely dispersed doses of grain, for the rearing and production phase of laying hens (Garrelfs, Hiller, Sagkob, & Diekmann, 2016; Keppler, Fetscher, Hilmes, & Knierim, 2017). The ‘Recommendation on the Prevention of SFP and Cannibalism’ in pullets and laying hens by the Ministry for Nutrition, Agriculture and Consumer Protection of Lower Saxony/Germany (Niedersächsisches Ministerium für Ernährung, Landwirtschaft und Verbraucherschutz 2017) also recommends the permanent provision of manipulable enrichment materials in addition to litter. According to this, enrichment material should be available in rearing systems with closed aviaries during the first weeks of life from the date of housing. Furthermore, this recommendation states that hens during the laying period must have permanent access to other changeable material, in addition to the litter, as this could significantly reduce the risk of SFP and cannibalism. Schreiter and Damme (2017) and Pottgüter et al. (2018), on the other hand, the recommendations do not provide clear advice on the permanent, preventive provision of enrichment material, except for friable scratchable litter. In the case of emerging SFP and/or cannibalism, however, the provision of additional enrichment material is unanimously recommended as a suitable countermeasure (Garrelfs et al., 2016; Keppler et al., 2017; Pottgüter et al., 2018; Schreiter & Damme, 2017).

## 5 CONCLUSIONS

Because SFP is based on foraging and feed intake behaviour, the provision of manipulable material in a husbandry environment is an approach that is intended to reduce the occurrence of SFP by the adequate exercise of these behaviours. As shown in the literature, a reduced effect on SFP of enrichment and litter substrate in a low‐complexity cage environment is evident. However, consistent results have not been obtained on the influence of additional enrichment material in littered housing systems, which represent the most common type of husbandry in Northwestern Europe. Thus, further research to investigate the effects of an additional supply of enrichment materials in barn husbandry on the occurrence of SFP and the biological performance in pullets and laying hens is strongly recommended. Of particular interest for deep‐litter barn systems are the following questions: (a) Can the incidence and the severity of SFP be reduced by permanent or transient offers of additional enrichment material? (b) What role does the provision of these materials play during the rearing period and in possible switches (addition or omission) between the rearing and laying period? (c) What effects can be expected on the biological performance and on the economics of egg production? and (d) What is the suitability of the different groups of enrichment material (objects without feed intake, substrates for feed intake, facilities with sand‐bath substrates) with regard to the effects on SFP and does a combined use of several substrates increase the effects on behaviour?

## CONFLICT OF INTEREST

The authors declare no conflicts of interest.

## ETHICS STATEMENT

This work is based on a review of the literature. The authors confirm that they have adhered to the ethical policies of the journal, as noted in the author guidelines for publication.
